# Seroprevalence of West Nile Virus in Tampa Bay Florida Patients Admitted to Hospital during 2020–2021 for Respiratory Symptoms

**DOI:** 10.3390/v16050719

**Published:** 2024-04-30

**Authors:** Emma C. Underwood, Iset M. Vera, Dylan Allen, Joshua Alvior, Marci O’Driscoll, Suzane Silbert, Kami Kim, Kelli L. Barr

**Affiliations:** 1 Center for Global Health and Infectious Disease Research, University of South Florida, Tampa, FL 33612, USA; emmau@usf.edu (E.C.U.);; 2 Morsani College of Medicine, University of South Florida, Tampa, FL 33612, USA; 3 Tampa General Hospital, Tampa, FL 33606, USA

**Keywords:** West Nile virus, seroprevalence, ELISA, Florida, emerging infectious diseases, COVID-19

## Abstract

West Nile virus (WNV) is an arbovirus spread primarily by *Culex* mosquitoes, with humans being a dead-end host. WNV was introduced to Florida in 2001, with 467 confirmed cases since. It is estimated that 80 percent of cases are asymptomatic, with mild cases presenting as a non-specific flu-like illness. Currently, detection of WNV in humans occurs primarily in healthcare settings via RT-PCR or CSF IgM when patients present with severe manifestations of disease including fever, meningitis, encephalitis, or acute flaccid paralysis. Given the short window of detectable viremia and requirement for CSF sampling, most WNV infections never receive an official diagnosis. This study utilized enzyme-linked immunosorbent assay (ELISA) to detect WNV IgG antibodies in 250 patient serum and plasma samples collected at Tampa General Hospital during 2020 and 2021. Plaque reduction neutralization tests were used to confirm ELISA results. Out of the 250 patients included in this study, 18.8% of them were IgG positive, consistent with previous WNV exposure. There was no relationship between WNV exposure and age or sex.

## 1. Introduction

West Nile virus (WNV) is an arbovirus that is primarily vectored by *Culex* mosquitoes, first isolated in Uganda in 1937 [1,2,3]. The disease arrived in the continental United States in 1999, was introduced to the state of Florida in 2001, and has now become endemic to North America [4]. Since the turn of the century, WNV has been responsible for a large number of avian deaths, primarily in crows, jays, and hawks, as the sylvatic cycle is between birds and mosquitos [1,2,5,6]. However, WNV antibodies have been detected in a variety of animals including mice, rats, bats, alligators, snakes, dogs, cats, and killer whales [7,8,9,10,11]. Severe disease and death occur in humans and horses as dead-end hosts [1].

Currently, WNV is considered the most common mosquito-vectored disease in the United States, having over 55,000 reported cases and almost 2500 reported deaths since 1999 [2,3,6,12]. Within the state of Florida, 467 cases have been reported since the disease’s arrival [12]. However, these numbers are likely a gross underestimation of WNV prevalence, as current detection of WNV in humans occurs primarily in healthcare settings when patients present with severe neurological manifestations of disease including acute flaccid paralysis, meningitis, or encephalitis [13]. Because the window of detectable viremia by RT-PCR has typically passed by the time patients seek healthcare, commercial IgM ELISA of CSF or serum are often used to diagnose WNV. Thus, it is very likely that most WNV infections never receive an official diagnosis and are undetectable by molecular testing [13].

The state of Florida currently uses sentinel chickens and mosquito surveillance and control to determine if WNV and Eastern Equine Encephalitis virus (EEEV) are present in local mosquito populations [14]. There are approximately 266 coops that are located throughout the densely populated areas of the state [4]. The surveillance data are used to create transmission models that direct public health and mosquito control efforts [14]. These flocks are viewed as ideal for non-human surveillance due to quick seroconversion, with anti-WNV antibodies being detectable only five days post infection, while maintaining low viremia that is unlikely to contribute to transmission [15,16]. As of 2020, 26 of 67 counties in Florida had sentinel chicken coops with chickens tested on a weekly basis [14,17]. In addition, there are 90 mosquito control programs in the state that perform mosquito surveillance [18]. Testing of mosquitos for arboviruses is performed by a single lab at the Florida Department of Health, and this vector surveillance program may not be sensitive enough to track ongoing transmission, which would trigger public health officials to institute more aggressive mosquito control efforts [18]. 

Nationwide surveillance for WNV disease has plummeted since 2006 due to decreases in federal funding allocations, leaving only 39% of the original budget [19]. This led to the highest prevalence of neuroinvasive cases of WNV in 2012, with 2873 cases and 286 deaths in the United States [19,20]. The SARS-CoV-2 pandemic saw further reductions in arbovirus surveillance, as most state and county labs were redirected to SARS-CoV-2 testing both in Florida and throughout the United States [18]. 

It is estimated that 80 percent of WNV cases are asymptomatic, with mild cases presenting as a nonspecific flu-like illness [3,6]. The Centers for Disease Control (CDC) estimate that about 1 in 150 people infected with WNV develop a neuroinvasive form of the disease, either with meningitis, encephalitis, or acute flaccid paralysis [3,6,21]. If a patient develops neuroinvasive symptoms, the mortality rate is around 10% [3]. 

The clinical effects of infection with WNV go beyond those of self-limited infection, with the rare incidences of encephalitis. Recent reports have shown that WNV can persist in the kidney for years following infection and has been linked with the development of chronic kidney disease [22]. Other studies have shown that patients who have been infected with WNV commonly experience depression, mood disorders, memory loss, and difficulties with activities of daily living for months to years post infection, leading to significantly reduced quality of life [6,21,23,24]. Despite this, there remains a lack of interest in the public health and research funding sectors to explore the true incidence and acute and chronic clinical impact of WNV infection. The true burden of WNV in humans residing in the United States is unknown, and WNV exposure could be a contributing factor to the rise in mental/cognitive health problems and kidney disease in the United States in the last 20 years.

The goal of this study was to measure the rate of WNV exposure in the Tampa Bay area by testing local specimens acquired during the COVID-19 pandemic. Additionally, we assessed if WNV exposure impacted the severity of COVID-19 disease upon hospitalization with flu-like symptoms. Severity was measured in length of hospital stay, ventilator use, and mortality.

## 2. Materials and Methods

### 2.1. Ethics Statement

Biobank protocols, patient clinical chart review, and sample procurement from discarded residual clinical samples were reviewed and approved by the Ethics Review Committee at the University of South Florida (USF IRB Study00085) in accordance with the 1964 Declaration of Helsinki.

### 2.2. Patient Samples

To test the prevalence of WNV in Tampa Bay, we tested previously collected human serum and plasma samples from admitted adult patients at Tampa General Hospital (TGH) during 2020 and 2021. Upon admission, patients were tested for COVID-19. Sex and age were recorded for all patients, while time to discharge (TTD), ventilator use, and mortality during hospital stay were recorded for COVID-19-positive patients. The control group consisted of patients admitted to TGH receiving inpatient care but testing negative for COVID-19. 

### 2.3. Serology

An indirect enzyme-linked immunosorbent assay (ELISA) tested for IgG antibodies in samples. The antigen for the assay was produced using Vero 76 (CRL-1587) cells inoculated with NY99 WNV (CDC) and incubated at 37 °C for three days. The virus was heat-inactivated at 55 °C for two hours and then stored at −20 °C until use. When conducting the ELISA, antigen was diluted in a 1:200 ratio of antigen to phosphate-buffered saline (PBS) in a 96-well plate and incubated overnight at 4 °C. The plates were blocked with 5% lamb serum and 0.01% Tween-20 in 1× PBS for one hour at 37 °C. A standard protocol of ELISA was conducted with samples diluted at a 1:100 ratio in blocking buffer and goat anti-human conjugated to horse radish peroxidase (HRP) secondary antibody at a 1:2000 ratio in blocking buffer [25]. Plates were developed with a tetramethylbenzidine (TMB) substrate kit (Thermo Scientific Cat#34021, Waltham, MA, USA), and ODs were obtained at 450 nm. Specimens with ODs valued greater than two times the mean plus two times the standard deviation of the negative control were considered positive for WNV IgG antibodies. The negative control was a pre-bleed non-human primate serum (BEI resources NR-41782). The positive control consisted of serum from a horse recently vaccinated for WNV, as goat anti-human HRP secondary antibodies reacts minimally with horse serum proteins.

Plaque reduction neutralization assays (PRNTs) are considered the gold standard confirmatory assay for serological exposure and were used in this study to validate all positive ELISA results following methods previously described [26]. The CDC recommends PRNT assays be performed on all positive ELISAs for WNV, even commercial kits, due to antigenic cross-reactivity with other flaviviruses important for human health [27]. Due to limited quantities of patient serum, a single dilution was performed of 1:100 for all serum samples, as it has been shown to be the minimum titer at which antibodies can provide protection against a subsequent infection [28]. This dilution also eliminates the matter of potential cross-reactivity which could be a concern at lower titers. WNV has been shown to cross-react with other flaviviruses at titers of 1:40 or less, which is much lower than the titers used for these assays [29]. St. Louis encephalitis virus (SLE) and Japanese encephalitis virus (JEV) are the main flaviviruses that cross react with WNV [30]. Both viruses are not endemic to Florida, but imported cases may occur. However, due to the dilution used in this study, cross-reactivity of these flaviviruses would be unlikely to occur [30]. 

### 2.4. Statistical Analysis

A cross-sectional design was used to determine WNV prevalence. To calculate odds ratios between WNV exposure and COVID-19 status, mortality, and ventilator use, a binary linear regression was used. Results were adjusted for sex and age. Odds ratios were calculated between sex and WNV status in a similar fashion, adjusted for age. An independent samples *t*-test was used to determine if WNV exposure impacted age and TTD. The results were deemed statistically significant at the *p* < 0.05 level. Samples with missing data were eliminated. The data analysis was performed using the Statistical Package for Social Sciences (SPSS) software by IBM, version 28 (Armonk, NY, USA).

## 3. Results

### 3.1. Patient Characteristics

Out of the included cases, 133 (53.2%) were female, 117 (46.8%) were male, and the average age of the patients was 53.37 years (SD 17.378) (Table 1). In addition, 121 patients tested positive for COVID-19, while 129 were COVID-19-negative (Table 1). Among the COVID-19-positive patients, 63 (52.1%) were female, and 58 (47.9%) were male, with an average age of 53.28 years (SD 17.999) (Table 1). Among the COVID-19-negative patients, 70 (54.3%) were female, and 59 (45.7%) were male (Table 1). The average age for this group was 53.30 years (SD 16.845) (Table 1). Of the 250 participants in this study, 110 (44.0%) were positive for WNV IgG antibodies after an ELISA was performed. Of these, 47 were confirmed positive, thus resulting in an 18.8% prevalence of disease exposure. 

An independent samples *t*-test showed nonsignificant relationship between age (*p* = 0.111) and TTD (*p* = 0.188) with WNV exposure. There was an increase in odds of WNV exposure in males when adjusted for age; however, the relationship was not significant (OR = 1.602, *p* = 0.152) (Table 2). Furthermore, 22.2% of males (*n* = 26) and 15.8% of females (*n* = 21) were positive for WNV.

To further explore the relationship between age and WNV exposure, age groups were formed by decades (0–19 years, 20–29 years, 30–39 years, 40–49 years, 50–59 years, 60–69 years, and 70 and up years). A chi-square test showed no relationship between age group and WNV exposure χ^2^(6, *N* = 250) = 10.135, *p* = 0.119 (Table 3).

### 3.2. WNV Exposure and COVID-19 Severity

A binary regression analysis showed no significant relationship of COVID-19 status (OR = 0.982, *p* = 0.956), mortality during hospital stay (OR = 1.044, *p* = 0.950), or ventilator use (OR = 0.967, *p* = 0.956) with WNV exposure when adjusted for sex and age (Table 4). 

## 4. Discussion

This study found that 18.8% of patient specimens had neutralizing antibodies for WNV, indicating that there is likely significant underreporting and underestimation of WNV disease. According to the United States Census Bureau, there are 1,513,301 residents of Hillsborough County Florida [31]. If this study’s prevalence estimates are extrapolated, 284,500 people in Tampa Bay alone would have been exposed to WNV, of which only 56,900 would experience any symptoms, and roughly 4896 would have had symptoms of neuroinvasive disease, whether or not they sought out healthcare. This is a significant discrepancy when compared to the 467 cases reported statewide by the CDC in the past 20 years [12]. 

This could be due to a variety of factors, including the self-limited nature of the disease, lack of training among healthcare providers on recognizing the symptoms of WNV, or even access to healthcare. Studies have shown that WNV infections are more prevalent in populations of lower socioeconomic status [32]. Regarding diagnosis, two retrospective studies found that only 37–40% of hospitalized patients with meningitis or encephalitis were tested for WNV [33,34]. This indicates that not only are tests underutilized, but most testing is conducted only in cases of rare neuroinvasive disease. Additionally, it is estimated that only 50% of individuals experiencing symptoms seek medical care, with only 5% of those ever receiving a WNV diagnosis [35]. However, even if 5% of symptomatic patients were diagnosed in Tampa Bay, using our suggested prevalence of 18.8%, it would result in around 14,225 diagnoses in Hillsborough County. 

While there are many causes for idiopathic instances of depression, confusion, memory loss, and mood disorder, WNV may be a contributing factor to the incidence of these symptoms, as they are long-term sequelae of WNV exposure [6,21,23,24]. Current estimates of depression among Florida citizens range from 14.7% to 18.0% [36,37]. These rates are similar to the incidence of depression in the United States [38]. Further, depression and other mental illnesses have risen in the past decade in both Florida and the United States as a whole [37,38]. While there has been a marked increase in cognitive and memory disorders in Florida and nationwide since WNV emerged in the United States, decreases in dementia and cognitive disorders have been reported in European Union countries like England and Sweden where WNV is absent [39,40]. The same trends have been reported for chronic kidney disease in the United States [41]. Of note, increases in chronic kidney disease have not been reported in countries like Norway and England where WNV is not endemic [41]. 

Present perception of WNV as a virus of low incidence and burden translates to a low perceived need and low financial incentive for pharmaceutical companies to develop treatments. However, if the seroprevalence of WNV in the United States is as high as this study suggests, in addition to the recent increase of cases across Europe in the past decade, then there is likely a need for a human vaccine [42]. Currently, there is no commercial vaccine for WNV in humans, and only one vaccine has made it to phase II testing, ChimeriVax-WN02 [43,44]. There are currently four equine vaccines licensed for use in the United States and include inactivated whole virus (Zoetis, Boehringer Ingelheim, Ingelheim am Rhein, Germany), recombinant canary pox (Merial), and inactivated flavivirus chimera (Merck, Darmstadt, Germany). There are licensed vaccines for five flaviviruses for humans, and the JEV live attenuated vaccine SA14-14-2 can induce a duration of immunity that lasts for several years after just a single dose [45]. JEV is closely related to WNV, and the viruses share significant sequence homology [43]. It is reasonable to conclude that a human vaccine for WNV could be produced with similar results. 

Until a vaccine is available, surveillance and screening for WNV should be increased. Further, because arboviral infections can cause significant post-acute infectious sequelae even in the absence of encephalitis, physicians should be educated about the importance of diagnosing WNV and other arboviral infections with and without neurological symptoms [46,47,48,49,50]. Our knowledge of WNV prevalence is only as good as the physicians diagnosing and reporting infections to local health departments and the CDC. Public health and vector control measures are based on these reports, and under-diagnosis and underreporting by healthcare providers could be putting communities at risk.

### Limitations and Future Directions

There are several limitations in this study. Firstly, time to discharge, mortality, and ventilator use were not recorded for all samples. Consequently, a meaningful comparison could not be made regarding disease severity upon hospitalization and WNV exposure. All samples were collected from TGH, and thus they are all cases severe enough to require hospitalization, and the trends found in this study may differ from those in the public as a result. To our knowledge, none of these patients were seen for WNV symptoms, nor were they tested for WNV by their providers.

## Figures and Tables

**Table 1:** Background characteristics of participants with a history of WNV exposure.

	COVID Positive	COVID Negative	Total
Sex			
Female	63 (52.1)	70 (54.3)	133 (53.2)
Male	58 (47.9)	59 (45.7)	117 (46.8)
Age			
Mean (Years) ± SD	53.38 ± 17.9999	53.39 ± 16.845	53.37 ± 17.378
WNV Status			
Positive	23 (19.0)	24 (18.6)	47 (18.8)
Negative	98 (81.0)	105 (81.4)	203 (81.2)
Total	121	129	250

SD = Standard Deviation.

**Table 2:** Odds ratio between sex and WNV status adjusted for age.

	Odds Ratio	95% Confidence Interval		*p*-Value
		Lower	Upper	
Female	1.00 (REF)	-	-	-
Male	1.602	0.841	3.055	0.152

**Table 3:** WNV exposure by age groups in years.

	WNV IgG Negative N (%)	WNV IgG Positive N (%)	Total N (%)
Age Group (years)			
0–19	4 (1.6)	1 (0.04)	5 (2.0)
20–29	13 (5.2)	7 (2.8)	20 (8.0)
30–39	28 (11.2)	4 (1.6)	32 (12.8)
40–49	33 (13.2)	5 (2.0)	38 (15.2)
50–59	45 (18.0)	13 (5.2)	58 (23.2)
60–69	38 (15.2)	14 (5.6)	51 (20.4)
70+	42 (16.8)	4 (1.6)	46 (18.4)
Total	203	47	250

**Table 4:** Odds ratio between Delta wave and control for WNV status adjusted for sex and age.

	Odds Ratio	95% Confidence Interval		*p*-Value
		Lower	Upper	
COVID-19 Status				
Negative	1.00 (REF)	-	-	-
Positive	0.982	0.518	1.862	0.956
Mortality				
Survived	1.00 (REF)	-	-	-
Deceased	1.044	0.271	4.027	0.950
Ventilator Use				
No	1.00 (REF)	-	-	-
Yes	0.967	0.295	3.174	0.956

## Data Availability

The datasets presented in this study are not readily available as the data are part of an ongoing study. Request to access the datasets should be directed to emmau@usf.edu.
